# Combined inhibition of PI3K and Src kinases demonstrates synergistic therapeutic efficacy in clear-cell renal carcinoma

**DOI:** 10.18632/oncotarget.25700

**Published:** 2018-07-10

**Authors:** Caroline Roelants, Sofia Giacosa, Catherine Pillet, Rémi Bussat, Pierre Champelovier, Olivier Bastien, Laurent Guyon, Valentin Arnoux, Claude Cochet, Odile Filhol

**Affiliations:** ^1^ Université Grenoble-Alpes, Inserm U1036, CEA, BIG-BCI, Grenoble, France; ^2^ Inovarion, Paris, France; ^3^ Université Grenoble-Alpes, Inserm U1038, CEA, BIG-BGE, Grenoble, France; ^4^ Centre Hospitalier Université Grenoble-Alpes, CS 10217, Grenoble, France; ^5^ Université Grenoble-Alpes, CNRS-CEA-INRA, Laboratoire de Physiologie Cellulaire et Végétale, Grenoble, France

**Keywords:** kidney cancer, synthetic lethality, 3D culture, protein kinase, targeted combinational therapy

## Abstract

Potent inhibitors of PI3K (GDC-0941) and Src (Saracatinib) exhibit as individual agents, excellent oral anticancer activity in preclinical models and have entered phase II clinical trials in various cancers. We found that PI3K and Src kinases are dysregulated in clear cell renal carcinomas (ccRCCs), an aggressive disease without effective targeted therapies. In this study we addressed this challenge by testing GDC-0941 and Saracatinib as either single agents or in combination in ccRCC cell lines, as well as in mouse and PDX models. Our findings demonstrate that combined inhibition of PI3K and Src impedes cell growth and invasion and induces cell death of renal carcinoma cells providing preclinical evidence for a pairwise combination of these anticancer drugs as a rational strategy to improve renal cancer treatment.

## INTRODUCTION

Most kidney cancers are renal cell carcinoma (RCC) including the major subtype clear cell RCC (ccRCC) which is notoriously refractory to traditional chemotherapeutic such as radiation and cytokine therapies. Since the new century, several agents targeting angiogenesis and signal transduction pathways such as sunitinib, temsirolimus, pazopanib have appeared, showing improved clinical benefit and survival in randomized prospective clinical trials. Yet, improvements are still required as many of the current therapies are limited by acquired resistance. The phosphatidylinositol 3-kinase (PI3K)/protein kinase B (Akt/PKB) pathway which is composed of multiple converging kinase cascades, is aberrantly activated in ccRCC [1–7]. Importantly, this activation observed in a large number of high-grade and high-stage ccRCC has been associated with adverse clinical outcome, suggesting that this pathway is a “target-rich” source for cancer therapy for which inhibitors may serve as effective therapeutic agents [2, 3, 6–8]. Consequently, more than 50 drugs inhibiting this pathway are now in clinical development. One of them is GDC-0941 (Pictilisib), an orally bio-available small molecule inhibitor that selectively targets all class I PI3K isoforms [8, 9]. Although GDC-0941 was well tolerated in preclinical studies in mice, and has entered phase II clinical trials [10, 11], the responses are short and patients frequently show disease progression within a year [12]. Indeed, PI3K activation in tumor cells is often accompanied by concurrent activation of other oncogenic pathways, explaining the de novo treatment resistance. Thus, targeting this multi-component pathway, either alone or in combination with other drugs is urgently needed [8, 13].

Overexpression of the epidermal growth factor receptor (EGFR) in metastatic ccRCC is associated with high tumor grade [14–16]. The non-receptor tyrosine kinase Src is an important downstream effector of the EGFR that is predominantly involved in invasion [16–21]. Its activation has been established as a poor prognostic factor in several types of cancers and its contribution to the appearance of malignant phenotypes in renal cancer cells has been reported [15]. In addition, Src regulates a wide range of cellular processes and is a common node of multiple resistance pathways suggesting it may be a useful target for therapy [18, 22, 23]. Saracatinib (AZD0530) is an orally available dual-specific inhibitor of Src and Abelson protein tyrosine kinases [24–26] which is currently evaluated in clinical trials [27]. Accumulating evidence suggest that Src interacts with and stimulates the PI3K/Akt pathway in cancer cells [28–31] and knockdown of PI3K was shown to inhibit Src activation suggesting a potential bidirectional crosstalk [32]. Here we show that PI3K and Src kinases are dysregulated in ccRCC tumor samples and may represent relevant therapeutic targets. Therefore, we hypothesized that simultaneous targeting of both pathways through pharmacologic inhibition might have synergistic effects on metastatic ccRCC. Mechanistic investigations of the PI3K inhibitor GDC-0941 in combination with the Src kinase inhibitor Saracatinib demonstrated synergistic inhibition of tumor cell proliferation, reduction of cell migration/invasion and pro-apoptotic signal induction in tumor cells grown as spheroids. These results were confirmed in treated organotypic short-term culture of tumor tissue slices. Finally, the drug combination efficiently inhibited tumor growth and induced apoptosis in an orthotopic xenograft model. Together, our findings provide preclinical proof of concept for a tractable target combination therapy in ccRCC.

## RESULTS

### Human renal carcinomas overexpress activated Src and Akt kinases

Sequencing of mRNA and clinical data extracted from the database TCGA for 523 ccRCC patients showed that higher levels of Src and Akt mRNA expression is strongly associated with adverse clinical outcome (Supplementary Figure 1). In particular, reduced Src (Supplementary Figure 1A) and Akt2 (Supplementary Figure 1B) transcript levels are individually associated with better survival rates.

Moreover, ten tumor samples characterized as ccRCC and their normal surrounding tissue were analyzed by Western blot (Supplementary Figure 2). Consistent difference in the expression of Akt protein could be observed between normal (NT) and tumoral (T) samples, whereas Src protein expression was often higher in non tumoral tissues. However, as visualized by the P-Src/Src and P-Akt/Akt ratios, 80% and 70% of tumor samples showed higher amounts of the activated forms of Src and Akt respectively, as compared to normal tissue. Moreover, 70% of these tumors showed a combined activation, suggesting that the PI3K and Src pathways may be upregulated in these tumor samples. Therefore, dysregulated Src and PI3K may represent relevant therapeutic targets that prompted us to evaluate the antitumor effects of GDC-0941 and Saracatinib as inhibitors of PI3K and Src kinases respectively.

### Renal cancer cells are sensitive to targeted PI3K/Src inhibition

**Figure 1:**
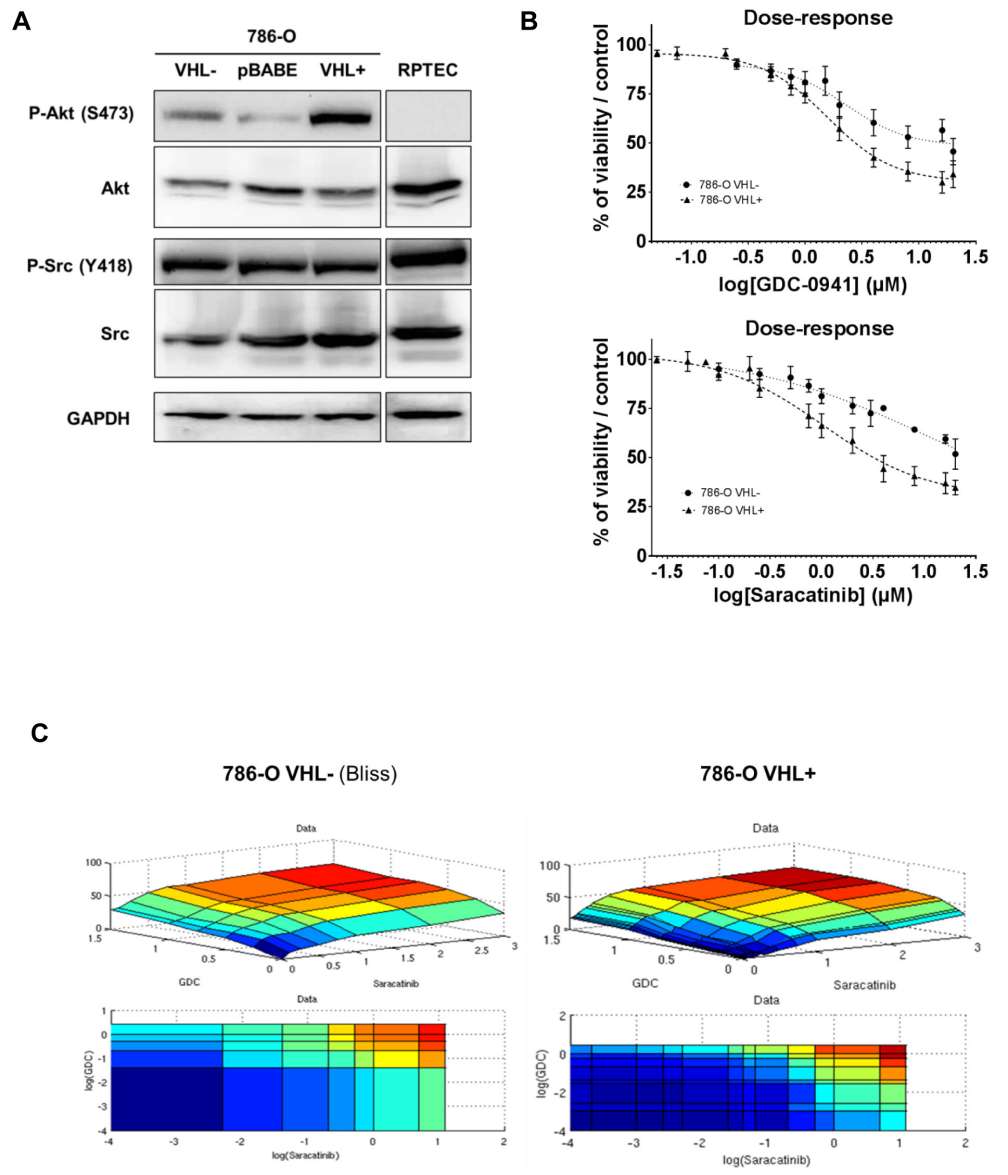
Protein kinases Src and Akt as targets in renal tissues and cell lines **(A)** Western blot analysis of WT (VHL^-^), Mock-transfected (pBABE), VHL-transfected (VHL^+^) 786-O cells and normal renal cells RPTEC. Both Src and Akt are detected for their expression and activity using anti total and phospho-site antibodies. GAPDH was used as a loading control. **(B)** Effects of Src and PI3K inhibition on the two 786-O cell lines. Cell viability of WT (VHL^-^; circle) and VHL-transfected (VHL^+^; triangle) 786-O cells was measured with Prestoblue^®^, upon dose-response of either GDC-0941 or Saracatinib for 48h. **(C)** Mathematical modelisation of dose-response matrix measurements on the two 786-O cell lines. Cell viability of WT (VHL^-^) and VHL-transfected (VHL^+^) 786-O cells was measured with Prestoblue^®^, upon dose-response of either GDC-0941 or Saracatinib for 48h. Data were processed to fit with three major models (HSA, Loewe and Bliss) that were evaluated by RMSE calculation.

We first evaluated the expression level of both Src and Akt proteins and their respective activated forms in the renal carcinoma cells 786-O VHL^-^ cells, and its derivate containing a functional VHL construct HA-VHL (786-O VHL^+^), together with a normal renal cell line (RPTEC) (Figure 1A, Supplementary Figure 3). Src protein was present and similarly active in all these samples. In contrast, activated Akt was absent in RPTEC and highly active in 786-O VHL^+^. We then tested GDC-0941 and Saracatinib on the growth and viability of 786-O VHL^-^ and 786-O VHL^+^ cells using the PrestoBlue assay. As single agent, GDC-0941 and Saracatinib inhibited ccRCC cell growth in a dose dependent manner (Figure 1B). However, we noticed that cell growth was partially impeded by either agents reaching only 40-50% inhibition at high concentrations, suggesting a potential compensatory mechanism (Figure 1B). 786-O VHL^+^ cells displayed higher sensitivity to GDC-0941 than 786-O VHL^-^ cells (GI_25_: 1.0 and 1.6μM respectively). Similarly, Saracatinib was more efficient on 786-O VHL^+^ cells compared to 786-O VHL^-^ cells (GI_25_: 0.6 and 3.2μM respectively). Treatment of 786-O VHL^-^ cells with increasing concentrations of GDC-0941 strongly decreased PI3K/Akt signaling, as evidenced by reduction in Akt phosphorylation. Surprisingly, this signaling was also downregulated at high concentrations of Saracatinib. As expected, Src phosphorylation was also inhibited in a dose-dependent manner by Saracatinib (Supplementary Figure 4A). Unexpectedly, in both cell lines, Akt and Src expression levels, were upregulated in response to either GDC-0941 or Saracatinib treatment respectively, suggesting a potential crosstalk between the PI3K/Akt and Src signaling pathways (Supplementary Figure 4A and 4B).

### PI3K and Src inhibition acts synergistically in 786-O cells

We hypothesized that combined inhibition of PI3K and Src that were moderately efficacious as single agents, could result in synergistic cell killing. Therefore, GDC-0941 and Saracatinib alone or in combination were tested on 786-O VHL^-^ and VHL^+^ cells at a concentration yielding 25% maximal response (EC_25_) by EC_25_ checkerboard design and evaluated by comparison of the experimentally derived impairment of cellular viability with the predicted combinatorial effect determined using the Bliss-additivity model [33]. When used as a single agent, GDC-0941 inhibited viability of 786-O VHL^-^ and VHL^+^ cells with IC_50_s equal to 4 and 1.6 μM respectively. Saracatinib demonstrated similar potency with IC_50_s of 3.5 and 1.4 μM respectively. Interestingly, a GDC-0941/ Saracatinib combination was highly synergistic in killing 786-O cells in the lower, clinically relevant dose range, reaching up to 40% more inhibition than predicted by Bliss additivity (Figure 1C). We also performed independent or combined inhibition of Src and PI3K/AKT pathways, using other inhibitors. The results show that Akt2 that is selectively inhibited by CCT128930, might not be involved as it had a low impact on 786-O cell mortality, and importantly, no synergistic effect in combination with Saracatinib (Supplementary Figure 5A). Moreover, other Src inhibitors like Dasatinib or Bosutinib which are more specific for Src, showed like Saracatinib, a synergistic effect in combination with GDC-0941 (Supplementary Figure 5B-5D). These results reinforce the assumption that simultaneous inhibition of PI3K and Src acted in a synergistic fashion to further impede 786-O cell growth *in vitro*.

### Simultaneous inhibition of PI3K and Src results in substantial induction of apoptosis

Immunoblotting of lysates prepared from VHL^-^ or VHL^+^ 786-O-treated cells showed that GDC-0941caused a marked induction of cleaved-PARP, a key substrate of activated caspases and an early indicator of apoptosis. This pro-apoptotic effect was significantly improved when both drugs were used in combination compared to single therapy. Similarly, cleaved Caspase-7 was strongly increased upon Saracatinib treatment. Again, this effect was potentiated when both drugs were used in combination (supplementary Figure 6).

### Combined inhibition of PI3K and Src results in decreased cell migration/invasion

**Figure 2:**
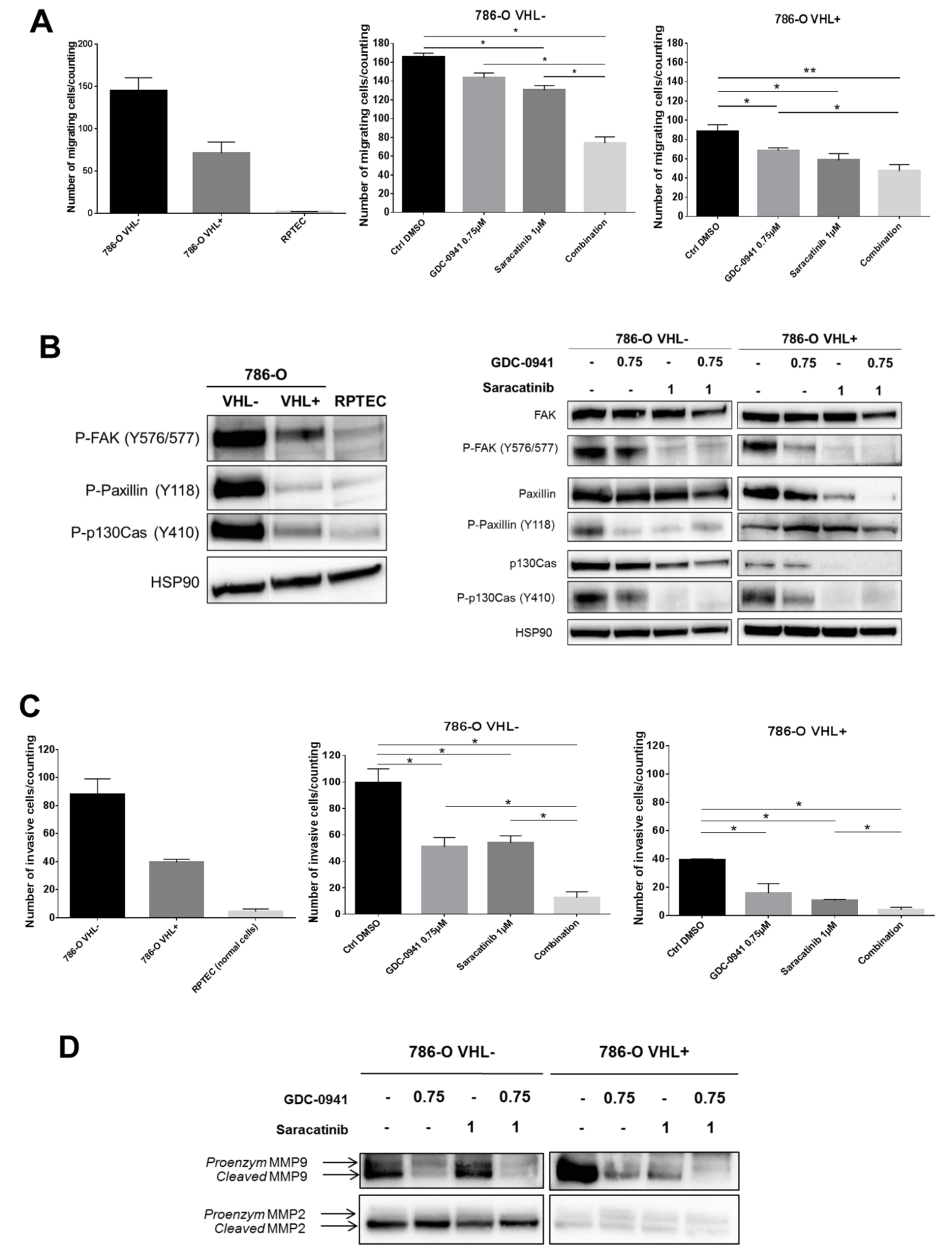
Mobility of renal cell lines **(A)** Transwell migration assay during 6h of indicated cells untreated (upper, left panel) or treated with 0.75μM GDC-0941 or/and 1μM Saracatinib (middle and right panels). Values were expressed as mean ± SEM and the statistical significance between 2 conditions was determined by Mann-Whitney test (n=4). **(B)** Western blot analysis of activated proteins involved in migration using whole cell lysates of the indicated untreated cell lines (left panel) and of 786-O-WT (VHL^-^) or expressing -VHL treated with either GDC-0941, Saracatinib or both (right panel) were probed with indicated antibodies. **(C)** Transwell Invasion assay during 24h of indicated cells untreated (left panel) or treated with 0.75μM GDC-0941 or/and 1μM Saracatinib (right panels). Values were expressed as mean ± SEM and the statistical significance between 2 conditions was determined by Mann-Whitney test (n=3). **(D)** Western blot analysis of metalloproteases using conditioned medium of the 786-O-WT or expressing -VHL treated with either GDC-0941, Saracatinib or both during 48h.

Migration and invasion are essential steps that have been shown to associate clinically with metastatic dissemination in ccRCC patients [15, 34]. Tyrosine phosphorylation of cellular proteins such as FAK, p130Cas and Paxillin plays critical roles in regulation of cytoskeleton and focal adhesion. Therefore, we first compared the migration properties of RPTEC and 786-O cells. As expected, RPTEC migration was almost undetectable. In contrast 786-O VHL^-^ cells exhibited exceedingly elevated migration as compared to RPTEC and 786-O VHL^+^ cells (Figure 2A, left panel). Accordingly, cell motility signaling components were differentially activated in 786-O VHL^-^, 786-O VHL^+^ and RPTEC cells as evidenced by phosphorylation of FAK (Y576/577), Paxillin (Y118) and p130Cas (Y410) (Figure 2B, left panel). We next assessed the effect of PI3K and Src inhibition on the migration of these RCC cell lines. Single-agent activity of GDC-0941 or Saracatinib at concentrations of 0.75μM or 1μM respectively, had marginal effects on the migration of either 786-O VHL^-^ or 786-O VHL^+^ cells. However, combined inhibition of PI3K and Src led to a significant synergistic inhibition of migration of 786-O VHL^-^ cells, whereas migration of 786-O VHL^+^ cells was weakly affected (Figure 2A, right panel). Single treatment of 786-O VHL^-^ cells with Saracatinib resulted in efficient suppression of Src autophosphorylation at Y418 (Supplementary Figure 4B) together with a strong decrease in the phosphorylation of FAK, Paxillin and p130Cas, which are signaling proteins required for efficient integrin-mediated focal adhesion formation (Figure 2B, right panel). The phosphorylation of these cytoskeleton proteins was also inhibited by GDC-0941 as a single agent (Figure 2B, right panel). These results show that both PI3K and Src pathways participate to the upregulated migration of RCC cells.

We next examined the inhibitory effect of GDC-0941 or Saracatinib on cell invasion using Boyden chamber assays. As expected, RPTEC invasion was almost undetectable in these assays. In contrast 786-O VHL^-^ cells showed exacerbated invasion properties as compared to 786-O VHL^+^ cells (Figure 2C, left panel). Single agent treatment with GDC-0941 or Saracatinib at a concentration of 0.75μM or 1μM respectively, had a significant inhibitory effect on the invasion of 786-O cells. Interestingly, invasion of both cell lines was almost completely suppressed by the GDC-0941/Saracatinib combination (Figure 2C, right panel).

The matrix metalloproteinases, MMP-9 and MMP-2, are upregulated in ccRCC promoting the ability to metastasize [35, 36]. We found that GDC-0941 caused in both VHL^-^ or VHL^+^ 786-O cells, a dramatic decrease in expression of MMP-9 in its cleaved and hence active form, whereas MMP-2 was not affected (Figure 2D). In VHL^+^ 786-O cells, MMP-9 activation was also affected upon Saracatinib-mediated Src inhibition and fully abrogated by simultaneous inhibition of both PI3K and Src pathways.

### Dual PI3K and Src inhibition promotes apoptosis on multicellular tumor spheroids

**Figure 3:**
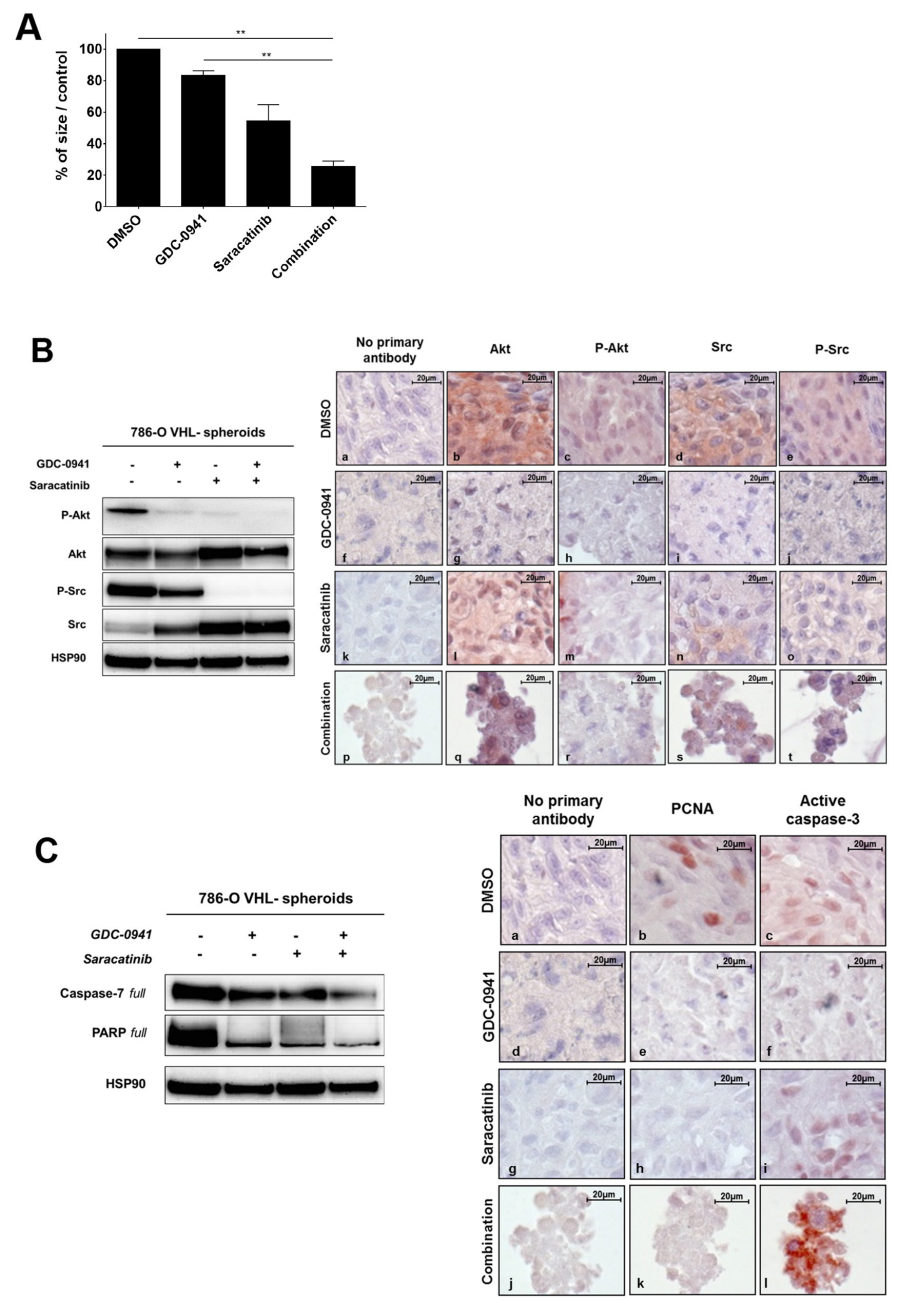
Spheroids **(A)** 786-O-WT (VHL^-^) cells were grown as spheroids andtreated with 20μM of either GDC-0941 (G20), Saracatinib (S20) or both (G20+S20) for 72h before measuring the spheroid size that is expressed as % of the size of the treated over the untreated spheroids (Ctrl DMSO). Values were expressed as mean ± SEM and the statistical significance between multiple conditions was determined by Kruskal-Wallis test (n=3). **(B)** The same spheroids treated with 20μM GDC-0941 or/and 20μM Saracatinib during 72h were analyzed by Western blot (left panel) or immunohistochemistry (right panel). Both Akt and Src were detected for their expression (b,g,l,q and d,i,n,s respectively) and activity (c,h,m,r and e,j,o,t respectively) using anti total and phospho-site antibodies. HSP90 was used as a loading control. **(C)** Apoptosis detection was performed on the same treated spheroids using both PARP and Caspase-7 with corresponding antibodies by western blot (left panel) or by immunohistochemistry (right panel) with PCNA (b,e,h,k) and Active Caspase 3 (c,f,i,l) antibodies.

The importance of studying cancer cells in three-dimensional (3D) models has been emphasized because of their higher relevance to *in vivo* situation [37–39]. To further explore the impact of combinational treatment of renal cancer cells, we analyzed cell growth and survival of 786-O VHL^-^ cells grown as 3D multicellular tumor spheroids (MCTS). After 72h of treatment with GDC-0941 or Saracatinib alone, a decrease in the size of the spheroids could be observed and this effect was more pronounced in response to the drug combination (Figure 3A). Western blot and immunochemistry analyses of these MCTSs showed that both GDC-0941 and Saracatinib were able to dampen the Akt and Src phosphorylation (Figure 3B, left panel). These effects were correlated with strong morphologic alterations of the spheroids (Figure 3B, right panel). Consistent with findings obtained with 2D cultures of 786-O cells, Akt and Src expression levels were upregulated in response to either GDC-0941 or Saracatinib treatment respectively, suggesting again a potential crosstalk between these two signaling pathways. We next assessed the effects of these drugs on the proliferation and apoptotic cell death in these tumor spheroids. GDC-0941 or Saracatinib as single agent induced significant levels of apoptosis visualized by cleavage of effector caspases-3/7 and PARP together with an inhibition of proliferation (PCNA staining). Moreover, the combined treatment led to a greater anti-proliferative and pro-apoptotic activity, resulting in massive morphologic alterations of MCTSs (Figure 3C, left and right panels).

### Validation of dual PI3K and Src inhibition on *ex vivo* explant cultures from renal tumor patient-derived xenografts (PDXs)

**Figure 4:**
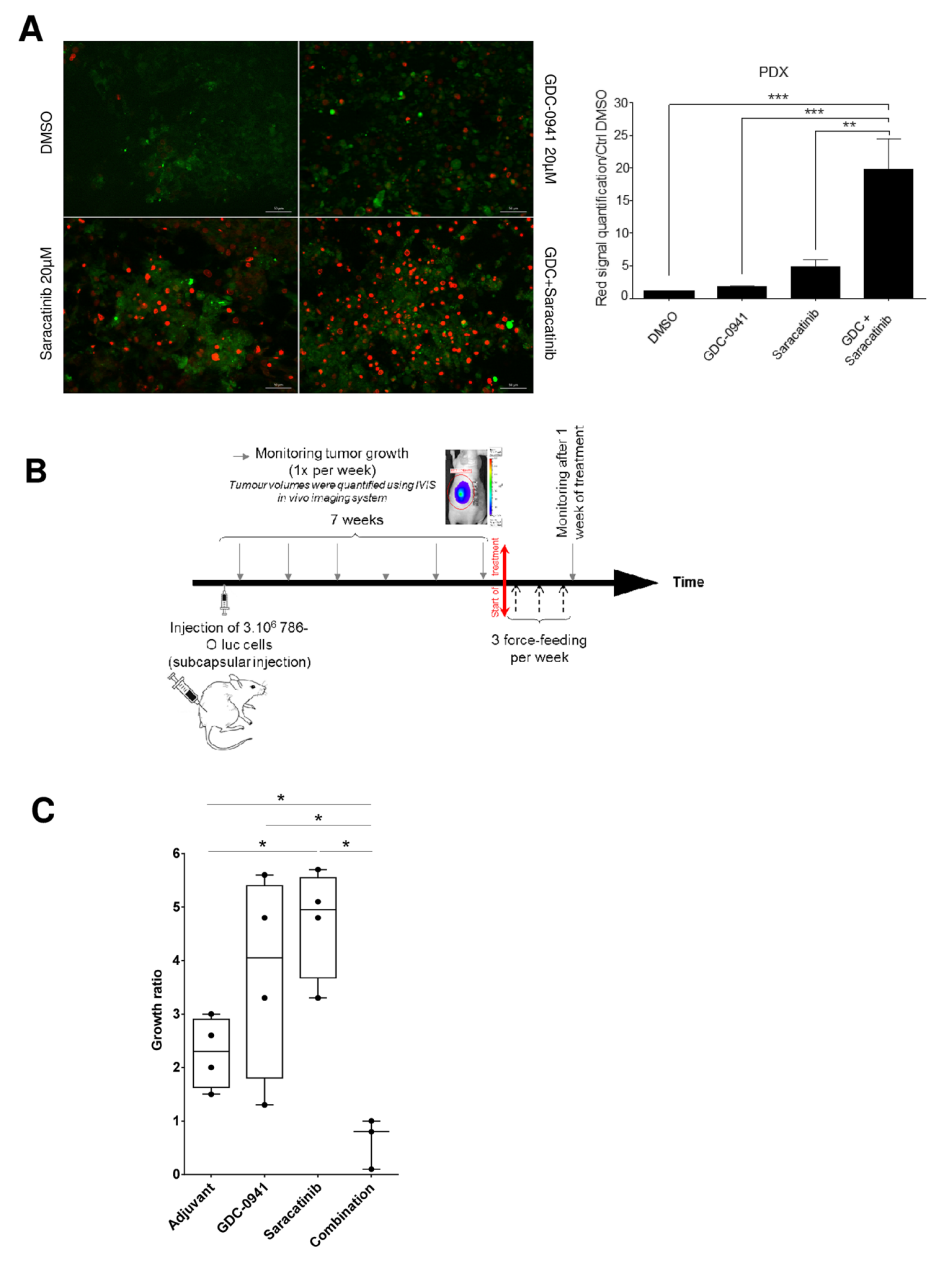
Tumor-suppressive effect of the combination **(A)** Tissue Slice cultures of a PDX RCC model were treated with either Vehicle (DMSO), GDC-0941 (20μM), Saracatinib (20μM) or a combination of both during 72h. Red marker intensity (Ethidium Bromide=dead cells) was measured on images taken with an Apotome-equipped Zeiss microscope. Right panel: Ratio of red fluorescence intensity compared to CTRL (DMSO). Significant difference was observed between GDC-0941 (^***^p≤0.001), Saracatinib (^**^p≤0.01) alone versus combination (Mann-Whitney test for all described conditions). Bar scale 50μm. **(B)** Time line of *in vivo* experiment on orthotopic xenograft model using 786-O Luc cells. Tumor size was monitored by IVIS imaging. 10mg/kg of Saracatinib or/and 100mg/kg of GDC-0941 were delivered via oral force-feeding, 3 times per week. **(C)** Growth measurement of the tumor. For each nude mice (4 per group), the ratio of the IVIS signal after over before treatment was calculated showing that combination treatment with GDC-0941 and Saracatinib induced tumor regression whereas chemicals alone promoted tumor growth. Values were expressed as min to max and the statistical significance between multiple conditions was determined (Mann-Whitney test).

Since PDX models more accurately recapitulate the clinical trial situation, the effect of Src and PI3K inhibition was evaluated on *ex-vivo* tumor slice cultures derived from one ccRCC PDX model. As illustrated in Figure 4A, left panel, Saracatinib and GDC-0941 to a lower extent, induced a detectable cell death that was strikingly enhanced by the drug combination. Again, quantification of dead cells highlighted that combining the two drugs resulted in increased synergistic apoptosis (Figure 4, right panel). Taken together, these results demonstrate that combined inhibition of PI3K and Src induces a massive cell death in tumor slice cultures derived from a PDX model.

### Orthotopic ccRCC xenografts show sensitivity to dual PI3K /Src inhibition

To test the effectiveness of inhibiting PI3K and/or Src *in vivo*, we performed orthotopic xenograft studies by injecting 786-O luc cells under the renal capsule. Drug administration started when average tumor size reached about 250 to 300 mm^3^. Using ccRCC vehicle-treated mice as a reference, mice were treated with GDC-0941or Saracatinib either alone or in combination every 48h for 7 days (Figure 4B). Vehicle and GDC-0941-treated 786-O tumor grafts showed similar growth rates, whereas unexpectedly, Saracatinib significantly increased tumor growth. In contrast, tumor graft growth was substantially inhibited by the drug combination relative to single-agent Saracatinib or GDC-0941 treatment (Figure 4C). Together, these data show that a combination of PI3K/Src inhibitors was more effective against RCC tumor grafts than Saracatinib or GDC-0941 treatment alone.

## DISCUSSION

Data from the literature suggest that both expression and activation of Akt and Src are associated with the appearance of malignant phenotypes and reduced survival in renal cell carcinoma [15]. Our data extracted from the TCGA database for ccRCC patients showed that high Src and Akt mRNA expression levels are strongly associated with adverse clinical outcome. In accordance, we showed an upregulation of activated Akt and Src kinases in ccRCC clinical samples. Activation of the PI3K/Akt/mTOR pathway in RCC was found to be associated with PTEN loss that occurs in approximately 30% of the cases [40]. Therefore, PI3K is an attractive target for therapy but resistance inevitably occurs as the result of feedback activation of other oncogenic signaling pathways, limiting the clinical utilization of PI3K inhibitors [12]. Akt over activity is hard to determine in patients [41] and most of the PI3K inhibitors are not isoform specific. However, it could be worth targeting Akt/PKB pathway in combination with an easily detectable biomarker such as Src activation [13]. We showed, in Supplementary Figure 1, that when overexpressed, both Akt1 and Akt2 are of poor prognosis whereas the data are at the opposite for Akt3. All the three Akt isoforms possess a Serine at position 473 that should be phosphorylated by PI3K. Thus, even if we can’t discriminate which of the three Akt isoforms is activated and targeted by our drug combination, the viability of the treated cells was strongly affected. We found that targeted inhibition of PI3K or Src in 786-O cells were moderately efficacious as single agents but showed enhanced and synergistic effect in combination. Moreover, both Akt and Src expression levels were upregulated in response to either GDC-0941 or Saracatinib treatment, suggesting parallel and compensatory targetable cross-activations in renal cell carcinoma. Several upstream effects of Src inhibition on the PI3K/Akt pathway have been reported. Src was shown to interact with and reduce the stability of PTEN resulting in an increase in the PI3K/Akt pathway [35]. PI3K inhibition promotes the induction of RTKs, suggesting that one of the drawback of PI3K inhibition is a hyper activation of RTK signaling thus explaining *de novo* treatment resistance. Indeed, we observed after GDC-0941 treatment a significant upregulation of Src targets involved in migration/invasion such as FAK, Paxillin and p130Cas suggesting the induction of an adaptive response. Migration and invasion are essential steps for metastatic dissemination of ccRCC and emerging evidence suggests that in cancer cells, Src is predominantly involved in invasion [16–21]. Blocking Src activity has anti-invasive and anti-migratory effects, inhibiting the growth of various types of cancers [36, 42–45]. Moreover, targeting Src and RTK simultaneously with Saracatinib and Sunitinib leads to synergistic inhibition of RCC proliferation and migration [16]. Thus, given its role as a common node of multiple resistance pathways, Src is an attractive target for combination therapies.

In this study, we showed that combined treatment with Saracatinib and GDC-0941exhibited greater-than-single-agent efficacy to promote vulnerability of RCC cells *in vitro*, impeding invasion and inducing cell death. Importantly, this combination was well tolerated, and caused marked tumor growth inhibition in ccRCC xenografts. As GDC-0941 and Saracatinib both exhibit excellent oral anticancer activity in preclinical models and have entered phase II clinical trials our findings, provide preclinical evidence for a promising pairwise combination of FDA-approved drugs with potent anticancer activity for further mechanistic study and translation to clinical trials.

## MATERIALS AND METHODS

### Reagent, drugs and antibodies

Antibodies against P-FAK (Y576/577) (#3281), P-Paxillin (Y118) (#2541), P-p130Cas (Y410) (#4011), PARP (#9542), Akt (#9272), Src (#2109), Active-Caspase-3 (#9664), P-Akt (S473) (#4051), Caspase 7 (D2Q3L) (#12827), VHL (#2738), MMP2 (#4022), MMP9 (#3852) and HSP90 (#4874) were from Cell Signaling Technologies (Danvers, MA). Antibodies against Paxillin (610052), FAK (610088) and p130Cas (610271) were from BD Biosciences (San Jose, CA). Antibody against P-Src (Y418) (LF-PA20465) was purchased from AbFrontier. Antibody against PCNA was from Abcam (Cambridge, UK). GAPDH (AM4300) was purchased from Life Technologies (Carlsbad, CA). Antibody against HA-12CA5 (11666606001) was from Roche. Dimethyl sulfoxide was from Sigma Aldrich (St Louis, MO). Saracatinib and GDC-0941 were obtained from LC Laboratories (Woburn, MA).

### Cell lines and culture

786-O cells, a highly metastatic cancer cell line which is null for VHL [46] were obtained from American Type Culture Collection (LGC Standards). RPTEC cells developed by overexpression of hTERT1 in normal renal proximal tubular epithelial cells were obtained from Evercyte (Germany). 786-O were grown in a humidified incubator (37°C, 5% CO_2_) with RPMI 1640 medium (Gibco) containing 10% fetal calf serum, penicillin [100U/mL], streptomycin [100μg/mL]. RPTEC were cultured in ProXup medium (Evercyte). 786-Oluc were generated by Optimal Platform (Grenoble). pBABE and VHL^+^ stable cell lines were obtained by transfecting 786-O with empty expression vector HA-pBABE or a functional VHL construct HA-VHL, respectively, obtained from Addgene. Stable transfectants were maintained in medium supplemented with 3μg/ml puromycin.

### Viability assay

Cytotoxicity was measured using the PrestoBlue^®^ assay (Invitrogen, Carlsbad, CA). Cell lines were seeded in 96-well microtiter plates at a concentration of 1×10^5^ cells/ml. Cells were allowed to attach for 24h at 37°C in 5% CO_2_. Cells were exposed to DMSO (negative control) or to Saracatinib or GDC-0941 alone or in combination with concentrations ranging between 0.1μM to 3μM and 0.25μM to 1.5μM respectively. The microtiter plates were incubated for 48h followed by the addition of 10μL PrestoBlue for 30min. The fluorescence was recorded at 580 nm using a FluoStar Optima plate reader (BMG LabTech, Ortenberg/Germany).

### Matrix analysis

Dose-response-matrix data were analyzed using the Highest Single Agent, the Loewe additivity and the Bliss models as described in Supplementary Material and methods.

### Migration and invasion assay

Cell migration and invasion assays were performed with 786-O VHL^-^, VHL^+^ and RPTEC cells. Cells were trypsinized, counted and resuspended in serum-depleted media (0,5% FBS), and plated at 2×10^5^ cells/well in 24-well Boyden chambers for migration assay (BD Biosciences), or at 5×10^4^ cells/well in 24-well Matrigel invasion chamber plates (BD BioSciences) using 10% FBS as chemoattractant. After 6h for migration assay or 24h for invasion assay, migratory or invasive cells were fixed with PFA and stained with Hematoxylin/Eosin. Cells within an entire field that migrated through the membrane were counted with a microscope.

### Spheroids culture

3-D cultures were carried out in 96-wells uncoated U-bottom tissue culture plates with low evaporation Lid (MicrotestTM, Becton Dickinson Labware, San Jose, CA) to allow the formation of multicellular spheroids. Renal cancer cells (786-O-VHL^-^ taken from exponentially growing cultures) were prepared at a density of 3×10^4^ cells/ml in EBM-2 medium (CloneticsR Lonza, Walkerville, MD) supplemented with 5% FCS, EGM-2MV (Lonza) and 0.2% (w/v) methylcellulose. One hundred μL (3×10^3^ cells) of this cell suspension were seeded in each well and the tissue culture plates were incubated at 37°C in 5% CO_2_ for 2 days before the addition of GDC-0941 and/or Saracatinib [47]. Drugs were prepared in EBM-2 medium at the desired concentration to give a final concentration ranging from 5μM to 30μM. Spheroids were grown in normoxic conditions and after 3 days of culture, the diameters of the spheroids were measured and the cell viability was evaluated using the PrestoBlue^®^ cell viability reagent. Spheroids were also washed twice with PBS and used for Western blot or immunohistochemistry analysis.

### Western blot analysis

Cell pellets were suspended in 150μl of lysis buffer (RIPA buffer: Tris HCl pH 7.4 10mM, NaCl 150mM, SDS 0.1%, Na Deoxycholate 0.5%, EDTA 1mM, Triton X100 1%, Protease and phosphatase inhibitor cocktails (P8340 and P5726, Sigma, St Louis)). Protein concentration was determined using the BCA protein Assay kit (Thermo Scientific). Protein extracts (20 μg/lane) were separated by electrophoresis on SDS-PAGE using pre-cast 4-12% gradient gel (Bio-Rad) at 150 volts for 75 min. Separated proteins were transferred onto polyvinylidene difluoride (PVDF) membranes (60 min at 100 Volts). Blotted membranes were blocked for 1h at room temperature in 1% BSA in TBST (50mM Tris-HCl (pH 7.5), 150mM NaCl, 0.2% Tween 20) and then incubated for 90 min at room temperature or overnight at 4°C with the appropriate primary antibody diluted in saturation buffer, followed by incubation with horseradish peroxidase (HRP)-conjugated secondary antibodies and detected by enhanced chemiluminescence. Anti-GAPDH or HSP90 were used as control for equal protein loading.

### Immunohistochemistry

Sections (5μm thick) of formalin-fixed, paraffin embedded tumor tissue samples were dewaxed, rehydrated and subjected to antigen retrieval in citrate buffer (Antigen Unmasking Solution, Vector Laboratories) with heat. Slides were incubated for 10min in hydrogen peroxide H_2_O_2_ to block endogenous peroxidases and then 30min in saturation solution (Histostain, Invitrogen) to block nonspecific antibody binding. This followed by overnight incubation with primary antibody at 4°C. After washing, sections were incubated with a suitable biotinylated secondary antibody (Histostain, Invitrogen) for 10min. Antigen-antibody complexes were visualized by applying a streptavidin-biotin complex (Histostain, Invitrogen) for 10min followed by NovaRED substrate (Vector Laboratories). Sections were counterstained with hematoxylin to visualize nucleus. Control sections were incubated with pool secondary antibodies without primary antibody.

### *In vivo* orthotopic tumor xenograft models

All animal studies were approved by the institutional guidelines and those formulated by the European Community for the Use of Experimental Animals. Six week-old BALB/c Female nude mice (Charles River Laboratories) with a mean body weight of 18-20g were used to establish orthotopic xenograft tumor models. The mice were housed and fed under specific pathogen-free conditions.

To produce tumors, renal cancer cells 786-O-luc (VHL^-^) were harvested from subconfluent cultures by a brief exposure to 0.25% trypsin-EDTA. Trypsinization was stopped with medium containing 10% FBS, and the cells were washed once in serum-free medium and resuspended in 500μl PBS. Renal orthotopic implantation was carried out by injection of 3×10^6^ 786-O luc cells into the right kidney of athymic nude mice. Seven weeks after the implantation of the xenografts, animals were randomly divided into treated and untreated groups. Mice were treated with vehicle (0.5% Tween 80), GDC-0941 (100mg/kg), Saracatinib (10mg/kg), or their combination by force-feeding, three times a week. Mice were weighed once a week to monitor their health and tumor growth was measured by imaging luminescence of 786-O-luc cells (IVIS).

A PDX model RCC-10-B was generated by Xentech, (Paris France). Briefly, tumor fragments (30mm^3^) were grafted in the inter-scapular subcutaneous tissue, one fragment per mouse. Tumors are metastasis in the lymph node of Clear cell/granular kidney Carcinoma. The status of VHL gene was determined as mutated (E160fs14aa (chr3-10191481-G->GA- (frameshift insertion) for RCC10B.

### Fresh tissue sectioning

A Vibratome VT1200 (Leica Microsystems) was used to cut thin (300μm) slices from fresh tissue. Samples were soaked in ice-cold sterile balanced salt solution (HBSS), orientated, mounted, and immobilized using cyanoacrylate glue. Slicing speed was optimized according to tissue density and type; in general, slower slicing speed was used on the softer tissues and *vice versa* (0.08-0.12mm/s neoplastic tissue; 0.01-0.08mm/s normal tissue). Vibration amplitude was set at 2.95-3.0mm.

### Organotypic tissue cultures

Tissue slices were cultured on organotypic inserts for up to 96h (one slice per insert; Millipore). Organotypic inserts are Teflon membranes with 0.4μm pores that allow preservation of 3D tissue structure in culture. Tissue culture was performed at 37°C in a 5% CO_2_ humidified incubator using 1ml of DMEM media supplemented with 20% inactivated FBS (GIBCO), 100 U/ml penicillin (Invitrogen) and place in a rotor agitator to allow gas and fluids exchanges with the medium. The tissue slices were incubated with the drugs at the indicated concentrations and after 72h, they were stained with the Live & Dead kit (Life technologies) as recommended. Images were taken with an Apotome-equipped Zeiss Axioimager microscope and dead cells were quantified using ImageJ.

### Statistical analysis

Experimental data are shown as mean ± standard error mean (SEM). Statistical analyses were performed using one-way analysis of variance (ANOVA) with multiple comparisons test (GraphPad Prism 6). A P value of less than 0.05 was considered to be statistically significant. ^*^P < 0.05, ^**^P < 0.01, ^***^P < 0.001 and ^****^P < 0.0001.

U Mann-Whitney tests were used to evaluate the significance of differential tumor growth and differential behavior between cell lines. The difference was considered significant when *P* was <0.05. Statistical analysis was performed using GraphPad, InStat Statistical Software, V6.00, San Diego, CA.

Survival plots were performed in R version 3.3.2 (ref. [48] using the survival library [49]. Data were generated by the TCGA Research Network (http://cancergenome.nih.gov/) and normalized and retrieved from OncoLnc [50].

## SUPPLEMENTARY MATERIALS FIGURES



## Abbreviations

ccRCC: clear cell renal cell carcinomas
PDX: Patient Derived Xenograft
FDA: Food and Drug Administration
EGFR: Epidermal Growth Factor Receptor
MCTS: multicellular tumor spheroid
PFA: Para-Formaldehyde
SEM: Standard error mean
VHL: von Hippel-Lindau
FAK: Focal Adhesion Kinase
RPTEC: Renal Proximal Tubular Epithelial cell
MMP: Matrix Metalloproteinase
3D: three dimensions
BSA: Bovine Serum Albumin
HRP: Horse Radish Peroxidase
