# Correction: Combined inhibition of PI3K and Src kinases demonstrates synergistic therapeutic efficacy in clear-cell renal carcinoma

**DOI:** 10.18632/oncotarget.26807

**Published:** 2019-03-15

**Authors:** Roelants Caroline, Giacosa Sofia, Pillet Catherine, Bussat Remi, Champelovier Pierre, Bastien Olivier, Guyon Laurent, Arnoux Valentin, Cochet Claude, Filhol Odile

**Affiliations:** ^1^ Université Grenoble-Alpes, Inserm U1036, CEA, BIG-BCI, Grenoble, France; ^2^ Inovarion, Paris, France; ^3^ Université Grenoble-Alpes, Inserm U1038, CEA, BIG-BGE, Grenoble, France; ^4^ Centre Hospitalier Université Grenoble-Alpes, CS 10217, Grenoble, France; ^5^ Université Grenoble-Alpes, CNRS-CEA-INRA, Laboratoire de Physiologie Cellulaire et Végétale, Grenoble, France

**This article has been corrected:** The correct author information is given below:

**Roelants Caroline^1,2^, Giacosa Sofia^1^, Pillet Catherine^3^, Bussat Rémi^1^, Champelovier Pierre^4^, Bastien Olivier^5^, Guyon Laurent^1^, Arnoux Valentin^4^, Cochet Claude^1^ and Filhol Odile^1^**

Original article: Oncotarget. 2018; 9:30066-30078. https://doi.org/10.18632/oncotarget.25700

