# Supplementary material for: Combined inhibition of PI3K and Src kinases demonstrates synergistic therapeutic efficacy in clear-cell renal carcinoma
Source: Oncotarget. 2018 Jul 10;9(53):30066–78. doi: 10.18632/oncotarget.25700 (PMC6059021; doi:10.18632/oncotarget.25700)
Supplement: Supplementary file 1 [file oncotarget-09-30066-s001.pdf]

Combined inhibition of PI3K and Src kinases demonstrates synergistic therapeutic efficacy in clear-cell renal carcinoma

SUPPLEMENTARY MATERIALS

Matrix analysis

Dose–response-matrix measurements produce a wide variety of response surface shapes that depend upon target connectivity in the disease network. For example, we can observe surface shapes that can be produced by dose-additive responses, and synergies can take the form of dose-shifts, effect boosts or coalisms. Such combination effects can be quantitatively described using parametric shape models of inhibition I(X,Y) as a function of the single agent concentrations X and Y, which are calculated in reference to the single-agent response curves I(X) and I(Y). Even if combination response shape models that describe many of the observed response morphologies, it should be noticed that in general the set of retained models is not orthogonal and is not a generator of all possible response morphologies.

We had tested three major models. (A) HSA, the activity of the Highest Single Agent, is a superposition of the X and Y single agent responses, calculated from the inhibitions I(X) at X and I(Y) at Y [1]. In mathematical terms, we have $HSA(X, Y) = \max(I(X), I(Y))$.

(B) Loewe additivity is the drug-with-itself reference for synergy, where ILoewe at (X,Y) yields additive doses relative to the components' effective concentrations X,Y at ILoewe.

Following Berenbaum and Lehàr [1], for each combined concentration (X,Y) we used an iterative approach to find the inhibition ILoewe that satisfies $(X/X_1) + (Y/Y_1) = 1$, where X_1 and Y_1 are the single agent effective concentrations. Starting with a guess that I= HSA, we interpolated the single agent curves to find X_1 and Y_1 that produce I, calculated the corresponding combination index CI, and used bisection to converge on a value of I with combination index CI=1 [1]. (C) Boosts in efficacy are quantifiable using a function based on the Bliss model for independent elimination probabilities [2], with a parameter b added to describe varying boost levels. More precisely, Bliss boosting is calculated by the following formula, $I_{Bliss} = I_X + I_Y + (\beta - E_{min})(I_X I_Y/(E_X E_Y))$ where Emin is the lesser of EX and EY, the limiting single agent efficacies. The one free parameter β, in units of effect, determines the amount of boosting above Emax, the greater of the single agent efficacies. Useful reference levels for Bliss boosting are 'cancelling', 'suppressing', 'masking', 'multiplicative' corresponding to Bliss independence, and 'saturating'. Of these models, only Loewe additivity has an a priori mechanistic basis. Information about the mechanism of an observed combination can be obtained by comparing an observed dose-response matrix (experimental data) to this set of models [1].

For each models, a root-mean-square error (RMSE) is computed. RMSE is a measure of the differences between values predicted by the model the values actually observed and is computed with the following formula,

$$\sum_{X,Y}\left(I_{observed}(X,Y) - I_{Model}(X,Y)\right)^2.$$

All computation has been done using the MATLAB software.

Quantitative real-time PCR

One microgram of total RNA prepared for the microarray hybridization was used to generate cDNAs by reverse transcription using the iScript system (Bio-Rad) as recommended by the manufacturer. Real-time PCR was performed using Bio-rad CFX96 apparatus and qPCR Master Mix (Promega). The values for the VHL gene were normalized to the U6. VHL forward and reverse primer sequences used are ATGGCTCAACTTCGACGGC and CCAGAAGCCCATCGTGTGTC, respectively.

Real-time PCR was carried out on C1000 Thermal cycler (CFX96 Real Time system, Bio-Rad) at 95°C for 10min, followed by 40 cycles of 95°C for 3s and 60°C for 30s. Data were analyzed with CFX Manager Software version V1.5.534.0511 (Bio-Rad). Normalized expression was calculated using the comparative CT method and fold changes were derived from the 2−ΔΔCt values for each gene.

REFERENCES

1. Lehar J, Zimmermann GR, Krueger AS, Molnar RA, Ledell JT, Heilbut AM, Short GF 3rd, Giusti LC, Nolan GP, Magid OA, Lee MS, Borisy AA, Stockwell BR, et al. Chemical combination effects predict connectivity in biological systems. Mol Syst Biol. 2007; 3:80. https://doi.org/10.1038/msb4100116.
2. Bliss WL. Early Man in Western and Northwestern Canada. Science. 1939; 89:365–6. https://doi.org/10.1126/science.89.2312.365-a.

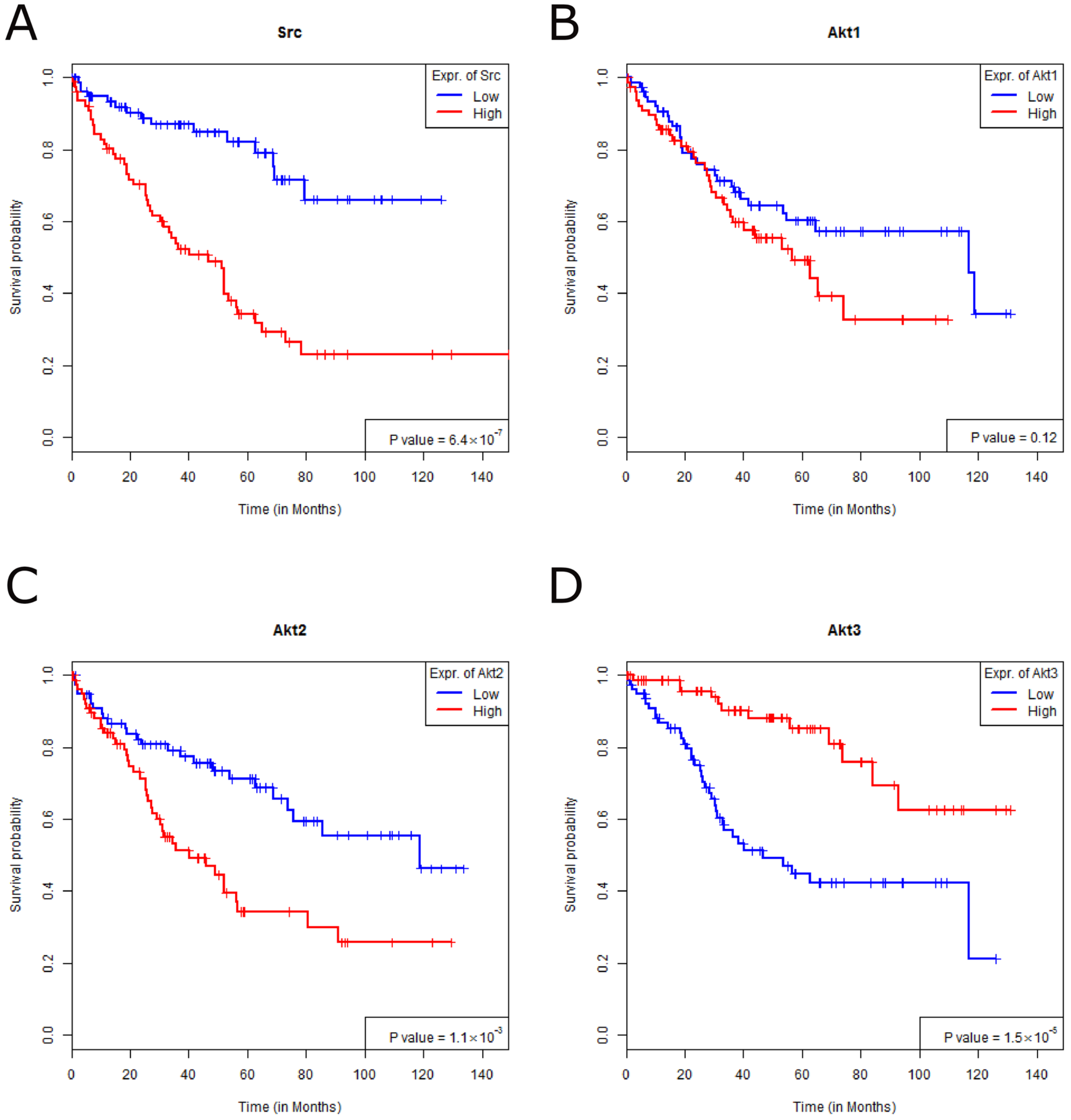

Supplementary Figure 1: Overexpression of Src and AKT2 is associated with lower overall survival in TCGA ccRCC patients. Kaplan-Meier estimates of overall survival. For each gene investigated, 2 equivalent groups of high (n=79 corresponding to last 15% quantile, in red) and low (n=79 corresponding to first 15% quantile, in blue) patients among 523 were defined. Src **(A)**, Akt1 **(B)**, Akt2 **(C)** and Akt3 **(D)** survival plots. P values of the log rank test are provided.

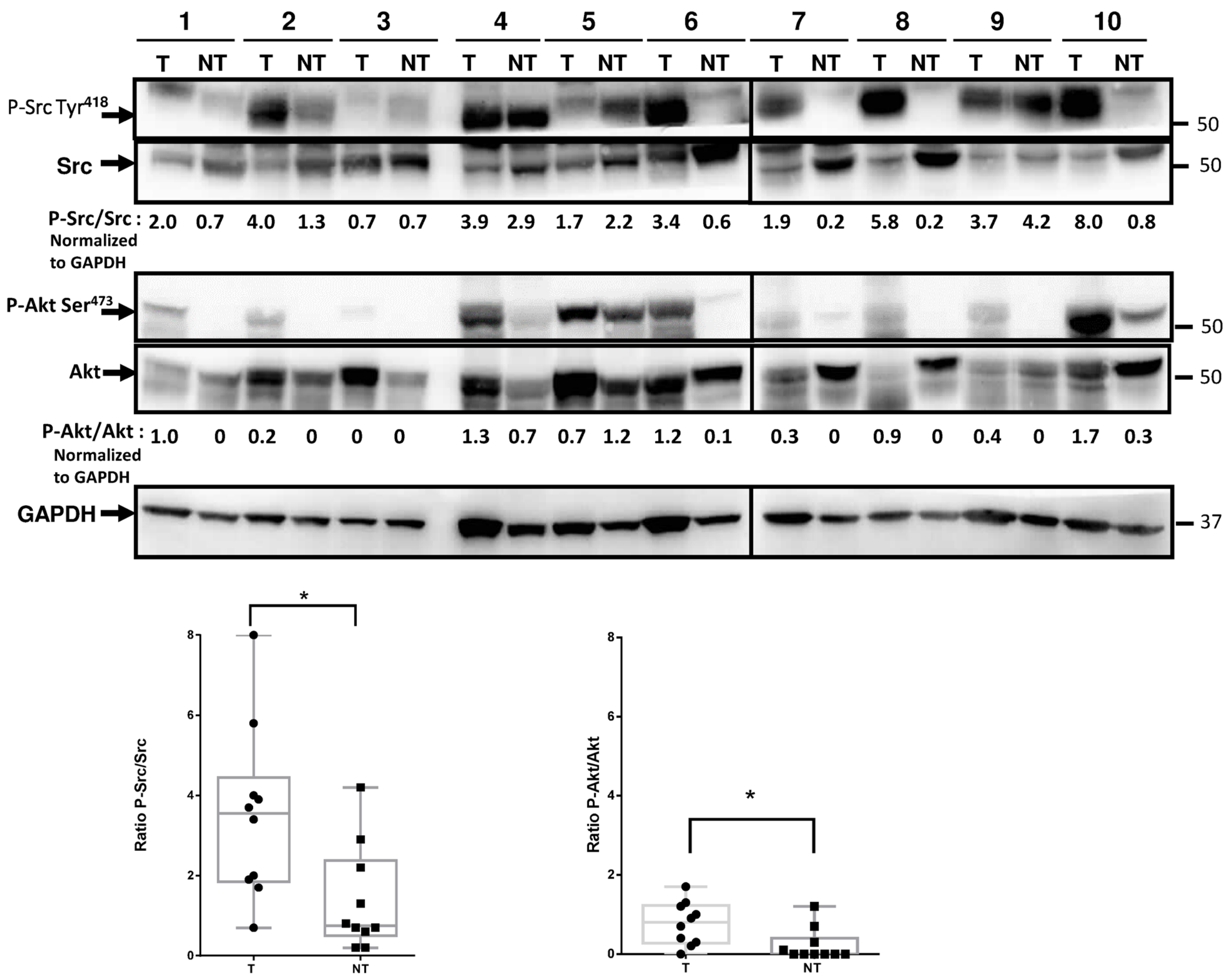

Supplementary Figure 2: Western blot analysis of Src and Akt expression and activity (phospho-antibodies) on a selected subset of 10 RCC samples (T) and adjacent normal tissues (NT). GAPDH was used as a loading control. Quantification of the bands was performed with ImageJ and ratio of phosphorylated versus total proteins were plotted. Significant difference was observed between T and NT samples (*p<0.015) (Mann-Whitney test).

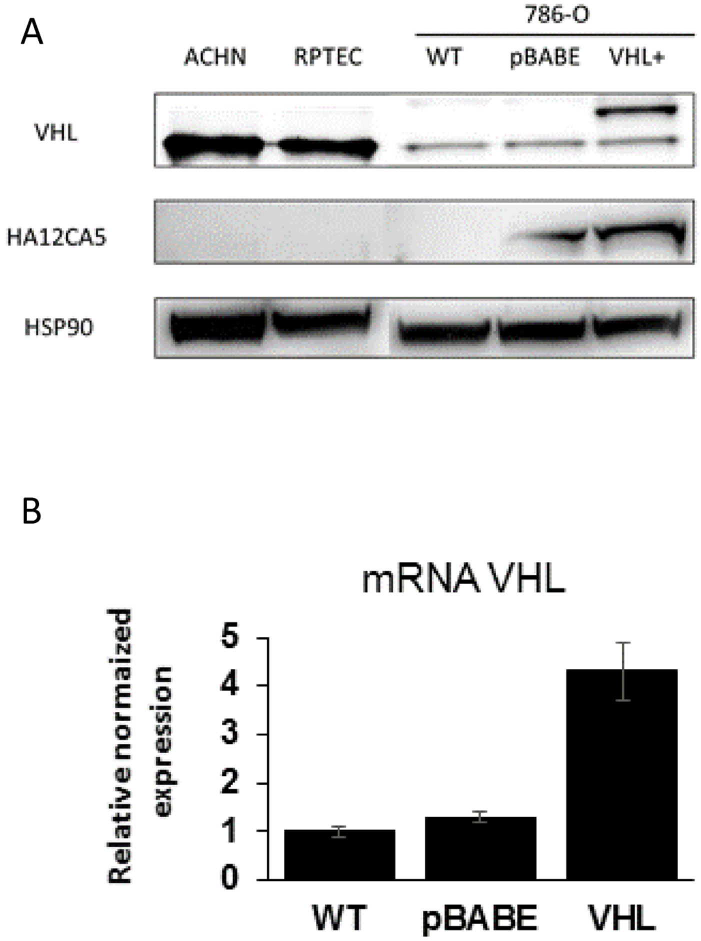

Supplementary Figure 3: Western blot and RT-qPCR analysis of VHL expression. (A) Indicated cell lines were analyzed by Western blot for their VHL protein expression using either VHL antibody or 12CA5 antibody that recognize the HA-tag of transfected VHL. HSP90 was used as a loading control. **(B)** RT-qPCR analysis of VHL mRNA in 786-O WT, pBABE- and VHL^{-} transfected cells.

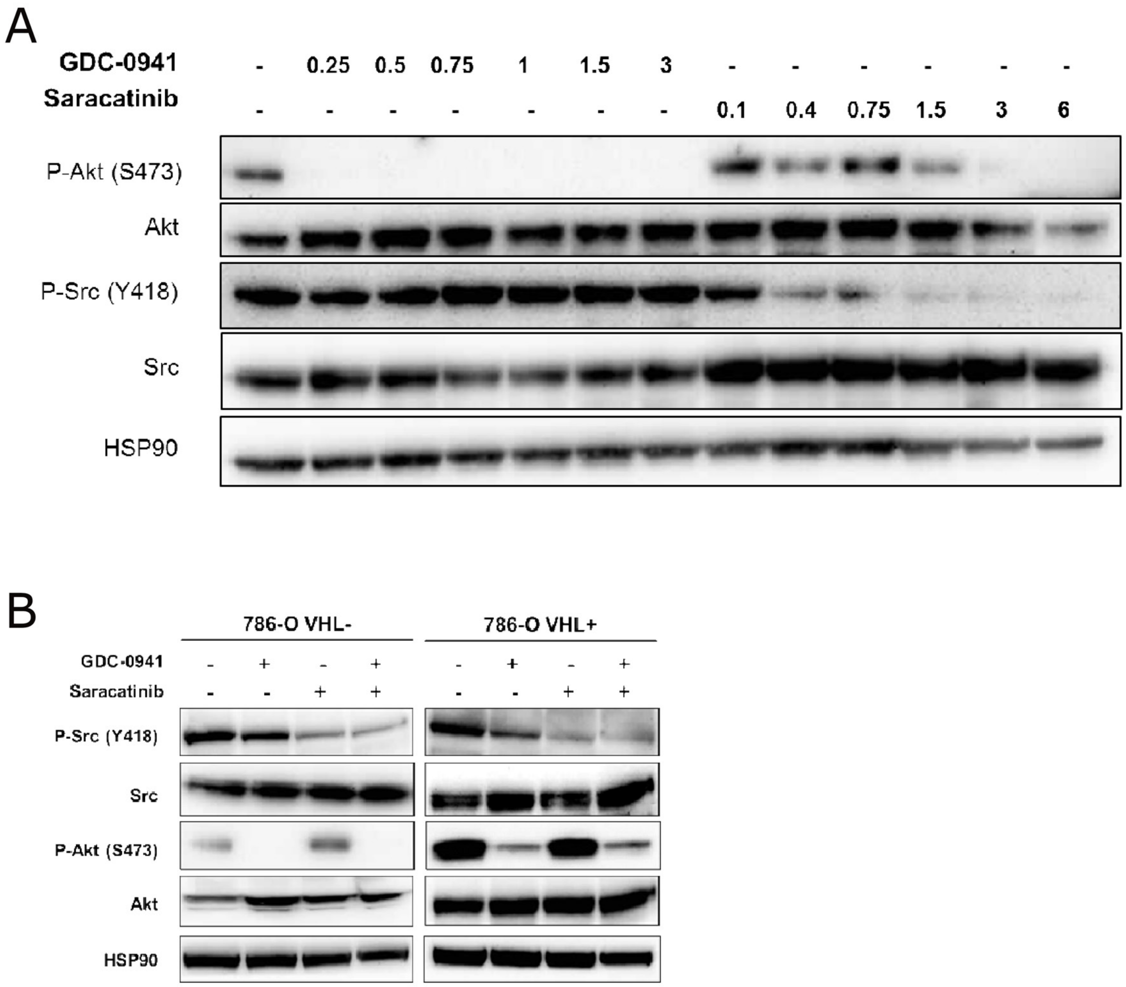

Supplementary Figure 4: Western blot analysis of Src and Akt expression and activity (phospho-antibodies). Both Src and AKT are detected for their expression and activity using anti total and phospho-site antibodies. HSP90 was used as a loading control. **(A)** 786-O VHL$^-$ cells treated with increasing concentrations of GDC-0941 or Saracatinib during 48h. **(B)** 786-O VHL$^-$ and VHL$^+$ cells treated with 0.75µM GDC-0941 or/and 1µM Saracatinib during 48h.

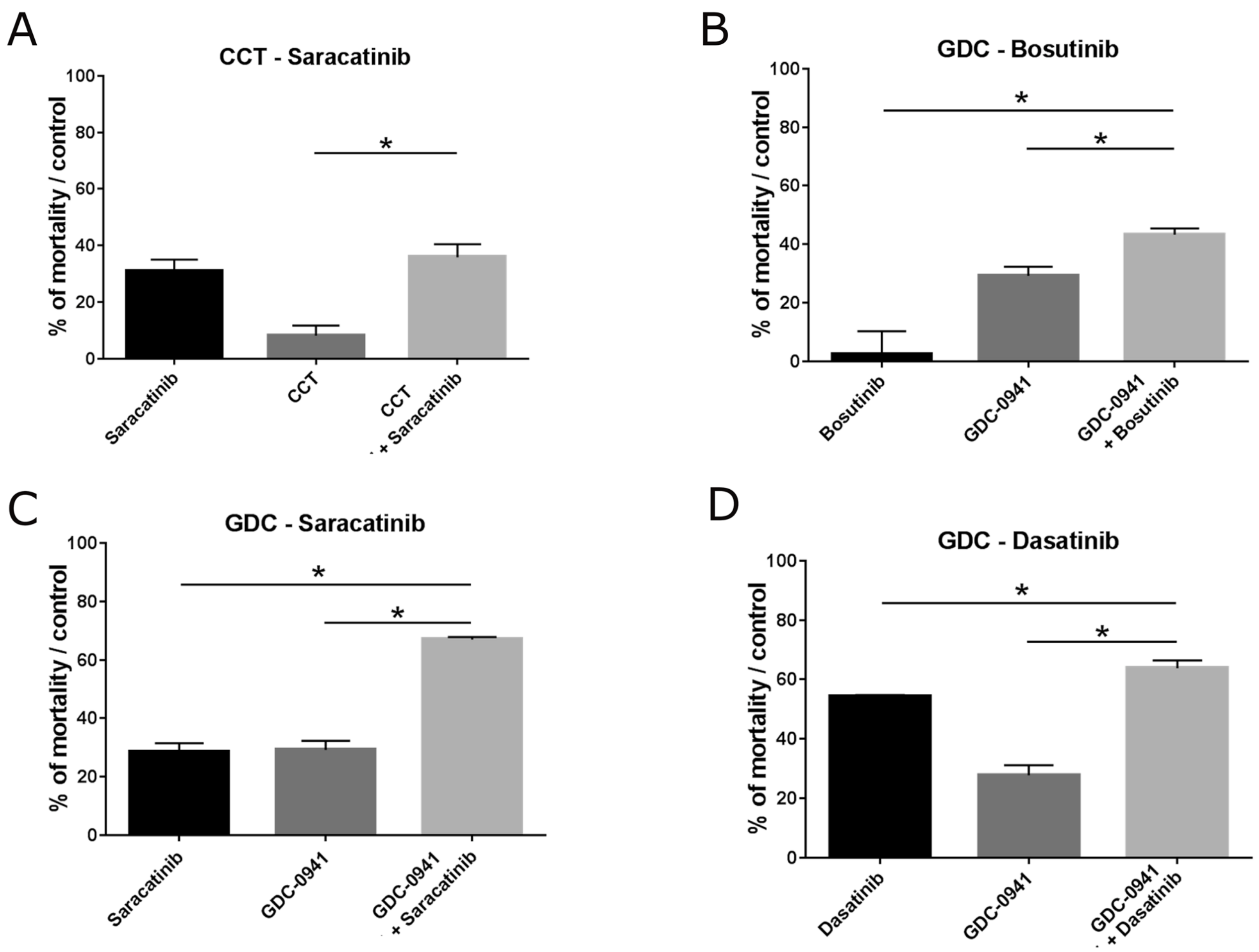

Supplementary Figure 5: Effects of Src and PI3K/AKT inhibition on the 786-O cells. Cell viability 786-O cells was measured with Prestoblue®, after treatment for 48h with inhibitors: **(A)** 1μM CCT128930 or/and 1μM Saracatinib; **(B)** 1μM GDC-0941 or/and 1μM Dasatinib; **(C)** 1μM GDC-0941 or/and 1μM Saracatinib; **(D)** 1μM GDC-0941 or/and 1μM Bosutinib. The percentage of mortality was calculated according to CTRL treatment (DMSO) as 0%. Significant differences are indicated, (*$p<0.05$) (Mann-Whitney test).

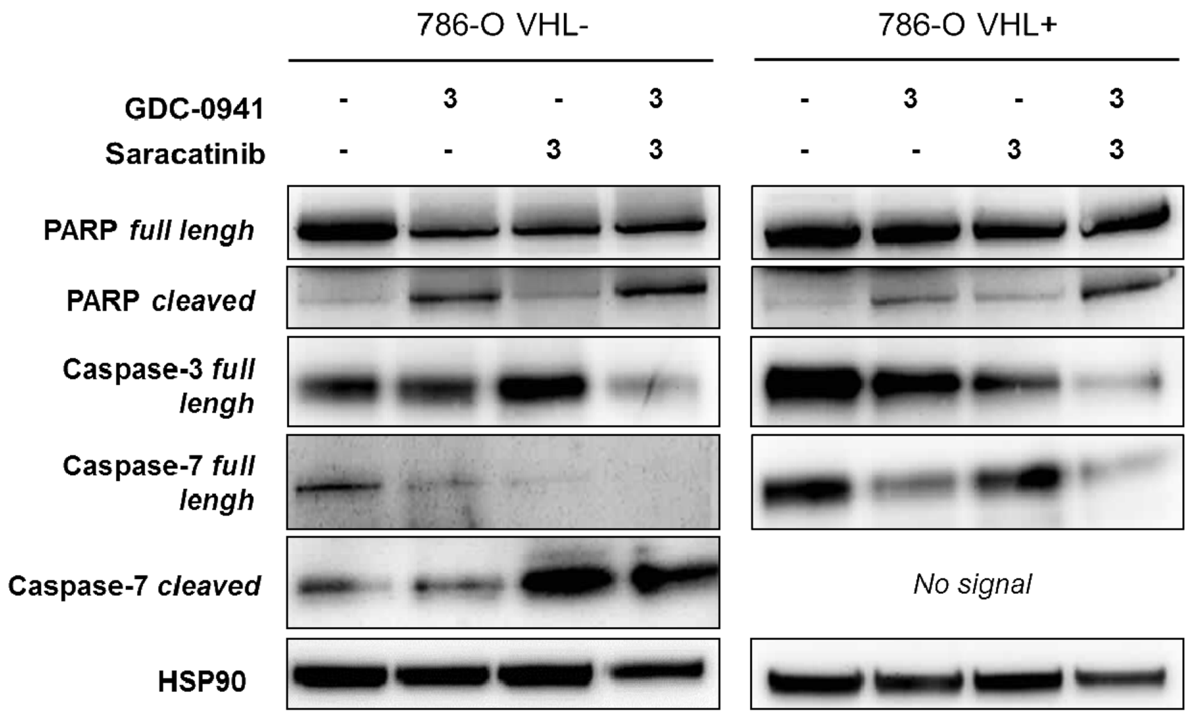

Supplementary Figure 6: Apoptosis. 786-O VHL$^-$ and VHL$^+$ cells treated with 3µM GDC-0941 or/and 3µM Saracatinib during 48h Apoptosis markers (PARP and caspase-3 and -7) were detected by western blot. HSP90 was used as a loading control.
